# Analysis of interactions between ribosomal proteins and RNA structural motifs

**DOI:** 10.1186/1471-2105-11-S1-S41

**Published:** 2010-01-18

**Authors:** Giovanni Ciriello, Claudio Gallina, Concettina Guerra

**Affiliations:** 1 Dept. of Information Engineering, University of Padova, Via Gradenigo 6a, 35131 Padova, Italy; 2 College of Computing, Georgia Institute of Technology, 801 Atlantic, Atlanta, GA, USA

## Abstract

**Background:**

One important goal of structural bioinformatics is to recognize and predict the interactions between protein binding sites and RNA. Recently, a comprehensive analysis of ribosomal proteins and their interactions with rRNA has been done. Interesting results emerged from the comparison of r-proteins within the small subunit in *T. thermophilus *and *E. coli*, supporting the idea of a core made by both RNA and proteins, conserved by evolution. Recent work showed also that ribosomal RNA is modularly composed. Motifs are generally single-stranded sequences of consecutive nucleotides (ssRNA) with characteristic folding. The role of these motifs in protein-RNA interactions has been so far only sparsely investigated.

**Results:**

This work explores the role of RNA structural motifs in the interaction of proteins with ribosomal RNA (rRNA). We analyze composition, local geometries and conformation of interface regions involving motifs such as tetraloops, kink turns and single extruded nucleotides. We construct an interaction map of protein binding sites that allows us to identify the common types of shared 3-D physicochemical binding patterns for tetraloops. Furthermore, we investigate the protein binding pockets that accommodate single extruded nucleotides either involved in kink-turns or in arbitrary RNA strands. This analysis reveals a new structural motif, called *tripod*.

It corresponds to small pockets consisting of three aminoacids arranged at the vertices of an almost equilateral triangle. We developed a search procedure for the recognition of tripods, based on an empirical tripod fingerprint.

**Conclusion:**

A comparative analysis with the overall RNA surface and interfaces shows that contact surfaces involving RNA motifs have distinctive features that may be useful for the recognition and prediction of interactions.

## Background

Protein-RNA interactions occur frequently within living cells as part of crucial processes, such as gene expression and regulation, protein synthesis and viral replication, as well as stabilizers of ribosomal RNA molecules within the ribosome. RNA recognition mechanisms raised much interest within the RNA community as soon as new structures became available. Several statistical studies were conducted on growing datasets of interacting structures [1-7], though few of them comprised a significant percentage of protein interactions with rRNA. The high resolution determination of complete ribosomal structures gave boost to protein-RNA interaction data availability, though it raised novel issues. First, ribosomal proteins (r-proteins) do not have previously observed homologs, they present unique features not present in other protein structures, such as the long unstructured tails that go deep into the ribosome. Second, r-proteins within a single species vary in structure, size and show tremendous diversity of interaction mechanisms even with similar RNA structural elements. Third, most interest has been devoted so far to the rRNA molecules as the main, probably the only, catalysts of the protein synthesis process. Thus r-protein have been less studied and considered only as stabilizers of RNA tertiary structure and involved in the subunit assembly. Indeed only recently the possibility of an effective involvement of r-proteins in the cellular processes performed by the ribosome has been explored [8].

A comprehensive analysis of ribosomal proteins and their interactions with rRNA has been done by [9] for the large subunit (*H. marismortui*, HM, 50S [10]) and by [11] for the small subunit (*T. thermophilus*, TM, 30S [12]). In [11] interesting results emerged from the comparison of r-proteins within the small subunit in *T. thermophilus *and *E. coli*, supporting the idea of a core made by both RNA and proteins, conserved by evolution. Though fascinating, the hypothesis is still difficult to validate due to the lack of high resolution data; indeed so far only four ribosomal high resolution crystals are available. On the other hand, in [9] authors analyzed the characteristic tails present within several r-proteins: these extensions, typically unstructured, show major presence of positive residues (arginine and lysine) and they often interact with RNA, even within the inner parts of the ribosome. Furthermore they proposed a classification of r-proteins within HM 50S based on secondary structure elements finding recurrent conformations. Similarly, attempts have been made to characterize RNA recognition sites from the protein secondary structure perspective in [13,2].

Recent work showed that ribosomal RNA is modularly composed [14-17]. Modules are structural motifs including bulge-free helices and conserved types of hairpin loops (e.g. tetraloops) and internal loops (e.g. kink-turns and loop-E motifs). Motifs are generally single-stranded sequences of consecutive nucleotides (ssRNA) with characteristic folding and, for some of them, sequence patterns.

The role of these motifs in protein-RNA interactions has been so far only sparsely explored. The only related work is in [18] where the kink-turn interaction mechanisms are studied, and in [19] and [20], where a characterization of the binding sites of ssRNA is provided. In particular in [20] the authors propose a 3D characterization of protein interaction sites with extruded dinucleotides, i.e. pairs of consecutive single-stranded nucleotides. Though common feature are highlighted, the results proved a high variability in terms of 3D conformation.

The goal of this study is to provide an insight into the role of RNA motifs on r-protein interactions, with a particular interest for tetraloops, kink-turns, and single extruded nucleotides. The analysis is conducted on two large ribosomal subunits, HM 50S (PDB no. 1S72) and the 50S subunit of E.coli (EC 50S, PDB no. 2AWB) and on the small subunit of TM 30S (PDB no. 1FJF). We will show how RNA motifs significantly interact with r-proteins, and analyze both composition and 3D conformation of interfaces made by motifs and proteins interacting with each other.

In the following, we will refer to RNA surface regions that are in contact with r-proteins as *RNA contact surfaces *(R-CS) and to the protein regions that interact with the RNA as *protein contact surfaces *(P-CS). We focus on the RNA interface regions consisting of atoms that participate in structural motifs. We denote such regions by SM_*R*_-CS. Similarly, protein contact surfaces restricted to those atoms that make contact with structural motifs are called SM_*P *_-CS. For the analysis, the RNA contact surfaces are extracted using the tool ENTANGLE [21].

## Result and discussion

The analysis on available crystal structures of ribosomes aims at uncovering relatioships, if any, between protein-RNA interaction mechanisms and RNA structural motifs. The goal is pursued first by quantifying the presence of RNA motifs in regions interacting with proteins, then by evaluating the composition of contact surfaces in the presence of motifs, and finally comparing and analyzing the 3D conformation of these surfaces.

### Frequency of Structural Motifs at interfaces

We are interested in establishing whether RNA structural motifs tend to appear more frequently at RNA interfaces than on the entire surface of the ribosome.

We start by briefly recalling the definitions of RNA motifs. A motif is composed by sequences, generally 1 or 2, of single-stranded consecutive nucleotides characterized by a distinctive 3D structure and, sometimes, sequence pattern. An RNA motifs is either a hairpin loop, i.e. connecting the two anti-parallel chains of one helix, or internal loop, i.e. connecting two helices. Known RNA motifs are: tetraloop, kink-turn, loop-E, *π*-turn, Ω-turn, and S2-turn. Definitions and methods to search for these structures are given in [14,22,17]. Complex loops connecting *k *≥ 3 helices and composed by *k *distinct ssRNA sequences are called RNA junctions. Junctions do not share conserved structures, but they show recurrent conformation in 3D [14].

The importance of motifs in protein-RNA interactions is testified by the high presence of these substructures within R-CS. In our analysis, each surface atom of the ribosomes is labeled as belonging to one of the following: bulge-free helix, known RNA motif, RNA junction, or other non-helical region. The frequency of atoms of different structural elements, presented in Table 1, is computed for the overall RNA structure, the entire RNA surface, and the RNA interfaces (col. 2,3,4 respectively). From the table we observe that in all three cases more than 50% of the atoms belong to non-helical regions and this percentage is almost identical (~52%) for the overall structure and the surface. By contrast, the distribution of atoms among RNA contact surfaces shows higher percentage of structural motifs to the detriment of helices.

**Table 1:** The frequency of atoms of different RNA elements in HM 50S is calculated for the whole 23S rRNA molecule (col. 2), for its surface (col. 3), and for RNA contact surfaces (col. 4). Motifs include tetraloops, kink-turns and other motifs such as loop-E motifs.

RNA element	23S (%)	23S surface (%)	RNA-CS (%)
Helices	48.6	48	44.2
Motifs	14.4	14.3	18.1
Junctions	13.8	16	15.7
Other non-helical regions	23.2	21.7	22

Major contribution to the high presence of motifs in R-CS is given by tetraloops and kink-turns: tetraloops are 4 residues long hairpin loops characterized by well conserved structure and consensus sequence patterns given by GNRA, UNCG, and CUUG, where N can be any nucleotide and R can be either G or A; on the other hand kink-turns consist of approximately 15 nucleotides from two distinct segments which base pair to form two helices and an internal loop.

However, the frequency of atoms of the different structural elements is not uniform across RNA interfaces with r-proteins For instance L18 extensively interacts with rRNA in both HM 50S and EC 50S, making ~50% of his interactions with structural motifs; by contrast L44e in HM 50S, L3 in EC 50S, and S4 in TT 30S, while forming large contact surfaces with rRNA, make no interaction with motifs.

### Interfaces composition

In this section we concentrate on the chemical composition of interfaces of H. marismortui, T. thermophilus and E. coli. In particular we will show that it exhibits remarkable differences depending on whether complete surfaces, interfaces, or interfaces involving structural motifs are considered. Variations are significant both at the RNA side, where we compare the distribution of phosphate-ribose-base (P-R-B) atoms, and at the protein side, where we analyze amino acid composition. These results are reported in Table 2, which also shows the bootstrap estimations of the standard errors [23].

**Table 2:** Composition of contact surfaces from rRNA of H. marismortui, T. thermophilus and E. coli.

Contact surface composition (% values)							
***RNA side***				***Protein side***			

	**R-CS**	**SM_*R*_-CS**	**TL_*R*_-CS**		**P-CS**	**SM_*P*_-CS**	**TL_*P*_-CS**
**P**	3.68 (± 0.32)	3.73 (± 0.70)	3.67 (± 0.87)	**Ala**	3.93 (± 0.50)	5.02 (± 1.25)	2.70 (± 1.27)
**OP1**	9.56 (± 0.76)	9.22 (± 1.43)	8.28 (± 1.23)	**Arg**	21.92 (± 1.67)	24.87 (± 4.08)	27.99(± 5.43)
**OP2**	5.61 (± 0.47)	5.79 (± 0.78)	5.00 (± 1.04)	**Asn**	4.41 (± 0.47)	3.40 (± 0.85)	3.07 (± 1.27)
**O5'**	3.20 (± 0.28)	2.99 (± 0.59)	2.92 (± 0.72)	**Asp**	2.58 (± 0.38)	2.53 (± 0.95)	1.42 (± 0.94)
**C5'**	9.43 (± 0.71)	8.95 (± 1.24)	9.79 (± 1.60)	**Cys**	0.19 (± 0.07)	0.16 (± 0.15)	
**C4'**	8.55 (± 0.65)	8.13 (± 1.09)	8.86 (± 1.35)	**Glu**	3.11 (± 0.36)	3.73 (± 0.88)	2.32 (± 1.09)
**O4'**	4.75 (± 0.39)	4.05 (± 0.60)	5.18 (± 1.01)	**Gln**	4.57 (± 0.61)	2.94 (± 0.78)	1.06 (± 0.88)
**C3'**	6.80 (± 0.53)	6.72 (± 0.97)	6.95 (± 1.09)	**Gly**	6.61 (± 0.80)	4.97 (± 1.25)	5.51 (± 2.38)
**O3'**	6.57 (± 0.55)	6.42 (± 0.89)	7.31 (± 1.34)	**His**	4.63 (± 0.53)	4.81 (± 1.14)	5.20 (± 1.81)
**C2'**	7.12 (± 0.52)	6.64 (± 0.85)	6.56 (± 1.18)	**Ile**	2.17 (± 0.25)	1.63 (± 0.50)	1.24 (± 0.75)
**O2'**	7.46 (± 0.56)	6.57 (± 0.91)	7.35 (± 1.25)	**Leu**	3.71 (± 0.38)	5.04 (± 1.13)	2.48 (± 1.91)
**C1'**	6.14 (± 0.46)	5.32 (± 0.64)	5.82 (± 1.02)	**Lys**	14.65 (± 1.06)	13.91 (± 1.97)	10.03 (± 2.01)
				**Met**	2.00 (± 0.31)	2.76 (± 0.75)	3.05 (± 1.87)
**P**	18.85	18.74	16.95	**Phe**	3.03 (± 0.41)	3.69 (± 1.05)	10.77 (± 3.18)
**B**	60.02	55.79	60.74	**Pro**	3.84 (± 0.56)	3.14 (± 0.82)	4.12 (± 1.94)
**R**	21.13	25.47	22.31	**Ser**	4.74 (± 0.52)	5.90 (± 1.35)	5.67 (± 2.42)
				**Thr**	4.67 (± 0.45)	2.75 (± 0.66)	1.77 (± 1.09)
				**Trp**	1.94 (± 0.38)	2.46 (± 0.98)	3.56 (± 2.07)
				**Tyr**	3.69 (± 0.49)	3.05 (± 0.86)	4.12 (± 1.73)
				**Val**	3.65 (± 0.47)	3.26 (± 0.79)	3.93 (± 1.53)

TL-CS are contact surfaces restricted to Tetraloops (R, P pedices indicate RNA and Protein side respectively). The bootstrap estimation of the standard error is shown in parentheses

#### P-R-B distribution

It is interesting to highlight how the proportions of phosphate, ribose, and base atoms vary in the protein-RNA complexes within the ribosome when they include structural motifs.

The results on complete interfaces of ribosomal RNA, reported in Table 2 (col. 2), partially confirm previous statistics on other RNA molecules [1,2,7]. First, an high percentage, approximately 80%, of backbone atoms, i.e. P and R, interacts with r-proteins. When we restrict the analysis to SM_*R*_-CS (Table 2, col. 3), such percentage decreases to ~75%. This was somewhat expected as RNA fragments that compose motifs include extruded nucleotides that typically interact through the exposed base. These results seem to follow the rule that double-stranded RNAs (dsRNA) interact with proteins mainly through the backbone, while ssRNA through bases [24]. Notable exceptions are tetraloops (Table 2, col. 4), where the percentage of atoms from phosphate and ribose groups rises again to almost 80%. This reflects the characteristic tendency of the tetraloop bases to form stacked conserved structures, and not to be exposed as typically ssRNA bases are.

In the ribosome, the ribose is preferred over the phosphate with a ratio of 3 to 1 for the whole contact surfaces and for the tetraloop contact surfaces, slightly below for contact surfaces restricted to RNA motifs. This fact is neither observable in protein-DNA complexes, nor in protein-RNA complexes not involving rRNA, suggesting that the high presence of the ribose group in protein-RNA interactions is distinctive of ribosomes[1,7].

What makes tetraloops favored motifs for protein interactions is also their position within the molecule: tetraloops are never completely buried. For instance in HM 50S only 4 out of 43 of them have less than 50% of atoms belonging to the surface, and backbone atoms are more exposed than the base ones, respectively 70% against 46% on average. The exposed position and the high number of instances favor these motifs in protein-RNA interactions within the ribosome: 43% of interactions within SM_*R*_-CS are indeed made by tetraloops. For all these reasons, tetraloops can be considered a good recognition motif for binding protein.

#### Residue composition

The composition of protein interfaces reflects known tendencies such as the high presence of arginine (Arg) and lysine (Lys) [11,9]. Both Arg and Lys are indeed positive residues that well connect to the negatively charged rRNA backbone. The percentages of all amino acids are shown in Table 2 (col. 5-8). Interesting variations are shown by TL_*P *_-CS when compared to both P-CS and SM_*P *_-CS. Tetraloops indeed show a significant preference for Arg, ~28%, over Lys with only 10%. The marked preference for Arg is justified by the large number of backbone interactions; for this reason the low presence of Lys is actually more surprising. Notable is also the presence of phenylalanine, Phe, (10.77%) that was not relevant within the whole contact surfaces. For TL_*P *_-CS Phe is significantly present in contact surfaces of both HM 50S and EC 50S, while less relevant in TT 30S. However in general the composition of these surfaces is diverse and the few atoms involved make the statistics highly dependent on single cases. This is showed by the relative high standard error in correspondence of most represented amino acids. Phe is for instance highly present in contacts made by L32e and L18 with HM 50S tetraloops, as well as those made by L2, L15, L18, and L34 in EC 50S; but scarcely present on the other contact surfaces with tetraloops.

### 3D conformation of interfaces

In this section we focus on the 3D conformation, localization and geometry of the protein interfaces with tetraloops, kink-turns and single extruded nucleotides. It has been observed [9] that strong similarities are not detectable in the overall shape conformations of the interfaces. This naturally follows from the unique structural features of r-proteins and from their flexibility. Thus the question arises of whether a significant conformational variability can be observed also in the more restricted areas binding well characterized motifs.

#### Interfaces with tetraloops

A significant fraction of r-proteins has a large number of contacts with tetraloops. In this section we examine the subset of r-proteins interacting with standard tetraloops in HM 50S as case study; thus the proteins considered are L2, L10e, L13, L14, L15, L15e, L18, L19e, L32e, L37e.

**Figure 1:**
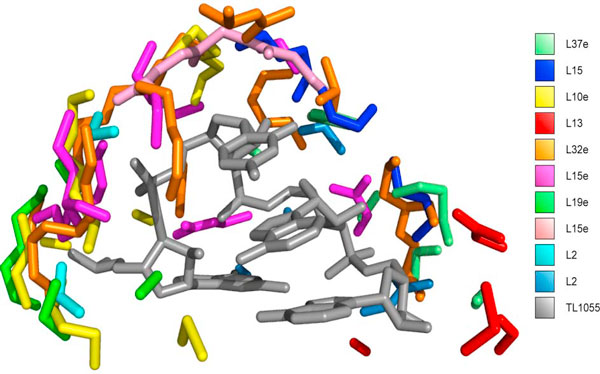
**The protein interfaces with tetraloops are displayed in a common frame defined by a reference tetraloop**. Each interface is different colored, the reference tetraloop is in gray.

The overall variation exhibited by SM_*P *_-CS is first assessed qualitatively by visual inspection of their localization with respect to their associated tetraloops. It is known [14,16] that standard tetraloops exhibit very similar 3D shape and, consequently, can be generally superimposed quite well. Thus, to visualize all TL_*P *_-CS we first superimposed the tetraloops and then transformed the interfaces accordingly. More specifically, all tetraloops are superimposed to single one, chosen as reference, using Horn's algorithm [25] applied to all corresponding backbone atoms of the tetraloops. For each tetraloop superposition the relative rototranslation was derived and the root mean square deviations (RMSD) computed. The obtained RMSD values are very small, in fact all values are below 1 Å. The rigid transformations are then applied to the protein interfaces. Figure 1 shows the localization of protein interfaces (each with a different color) with respect to the chosen reference tetraloop (in gray). From the figure it can be observed that the interfaces occupy different areas surrounding the backbone of the reference tetraloop and that they have different size and shape.

#### Area of the interfaces

**Figure 2:**
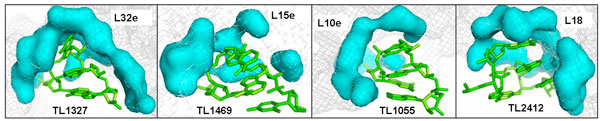
**The four most extended tetraloop contact surfaces show high shape complementarity**. In the figure TL1327 interacts with L32e, TL1469 with L15e, TL1055 with L10e, and TL2412 with L18.

For a more quantitative comparison, we determined the size of the interfaces measured both by the number of superficial atoms that compose them, and by their area. The contact surface area has been computed using the ^3^V web tool by [26]http://www.molmovdb.org/cgi-bin/3v.cgi, by rolling a probe sphere (*r *= 1.5 Å) on the contact surface. Table 3 shows the size of tetraloop contact surfaces of r-proteins along with the interacting tetraloops and their nucleotide sequence. A wide range of values for the areas can be observed from the Table 3. The largest contact surfaces are shown in Figure 2 together with the interacting tetraloop. The extended tetraloop contact surfaces make major interactions with the backbone, though with a less pronounced tendency than the whole set of tetraloop contact surfaces. The extensive interaction area in these cases comprise interactions with most of the atoms of the motifs, including the bases. This is notable for L32e and especially for L18, that is indeed the only surface that significantly interacts with the three stacked bases of TL2412. Surfaces formed by L2, L13, L15, L37e, L19e, and L15e with TL1863 are relatively small; these proteins interact preferably with two or three nucleotides, and generally with their backbone atoms. These contact surfaces neither share conserved 3D conformation, nor show preferences towards specific nucleotides.

**Table 3:** The protein-RNA complexes made by r-proteins and standard tetraloops.

Tetraloop contact surface				
**r-protein**	**Tetraloop**	**Sequence**	**Area (Å^2^)**	**Atoms No.**
L2	TL2249	GGGA	117.5	8
L15e	TL1863	GCAA	127.8	14
L15	TL691	GAAA	139.5	15
L13	TL1238	CGGG	155.2	14
L2	TL2630	GUGA	174	13
L19e	TL1794	GGAA	188	15
L37e	TL469	GUGA	257.25	24
L10e	TL1055	GUAA	375.4	34
L15e	TL1469	CAAC	401.9	40
L32e	TL1327	GAAA	552.5	63
L18	TL2412	GAAA	580.3	58

#### Interaction Maps

**Figure 3:**
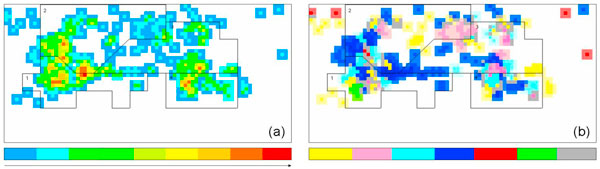
**Interaction map of all protein interfaces with tetraloops (a) atom distribution; the color scale goes from blue to red following the increasing atom density (number of atoms within a cell)**. The polygons with black perimeters mark the regions corresponding to the backbone of the tetraloops, labeled 1 to 4 specifying the position of the nucleotides (b) The interaction map is colored according to the amino acid physico-chemical properties: yellow is hydrophobic, pink aromatic, cyan polar, blue positive, red negative, green proline. Gray is used for gap cells. Colors are more intense where the density is higher.

For ease of visualization and analysis we built a two-dimensional representation of contact surfaces, that we called *interaction map*. Interaction maps basically map in a discretized 2D space the positions of the atoms of all TL_*P *_-CS after they are brought into a common reference frame (see Methods). Figure 3 shows the interaction map computed for all TL_*P *_-CS in HM 50S. Figure 3(a) visualizes the atom density distribution after all interfaces are mapped into the map.

To better study the localization of interactions between r-proteins and tetraloops, in the interaction map we mark the regions corresponding to the discretized coordinates of all tetraloops atoms after superposition with the reference. We identify four connected non overlapping regions corresponding to the backbone atoms of the 4 nucleotides of all tetraloops; they are labeled with numbers 1,..,4 and are shown by depicting their perimeter (in black) in Figure 3. Because of the rigidity of the tetraloops, the 4 regions are well separated (as can be seen from the figure). The following analysis does not include L18 interface with TL2412. As already pointed out, this interface represents the only exception making most interactions with the bases of the tetraloop.

The interaction map allows to appreciate the common tendency of the proteins to interact with tetraloop backbone atoms: the vast majority of protein interface atoms is located within the perimeters of the four regions and only a few cells outside the four regions are populated. Note that a preference is shown towards the area between the first and the second nucleotide and between the third and the fourth nucleotide, where the density of the atoms is higher.

Next, the interaction map is used to visually identify patterns of interactions in terms of physico-chemical properties of the residues. A color code of such properties has been applied to the interaction map. Since a cell of the interaction map may contain several atoms, a majority rule has been used. Precisely if most of the atoms within a cell have the property *p*_*i*_, than the cell is attributed to property *p*_*i *_and colored according to the code; else if a majority is not detectable, the cell is considered as a gap in the pattern. Figure 3(b) displays physico-chemical properties in the interaction map.

The resulting pattern shows a concentration of positive residues, mainly Arg, in the dense area between the first and the second nucleotide where the rRNA chain has a turn, changing the base orientation, to form the loop. High presence of Arg is also detectable between the third and the forth nucleotide, in correspondence of the other dense area. The same patches also contain several polar residues (especially Ser), while hydrophobic residues are more uniformly located within the surfaces.

#### Interfaces with kink-turns and single extruded nucleotides

The authors of [18] were the first to observe that kink-turns are an important RNA recognition motif for the r-proteins of the large ribosomal subunit. They identified a common interaction with the extruded nucleotide, observed in 4 out of the 6 double-stranded kink-turns.

**Figure 4:**
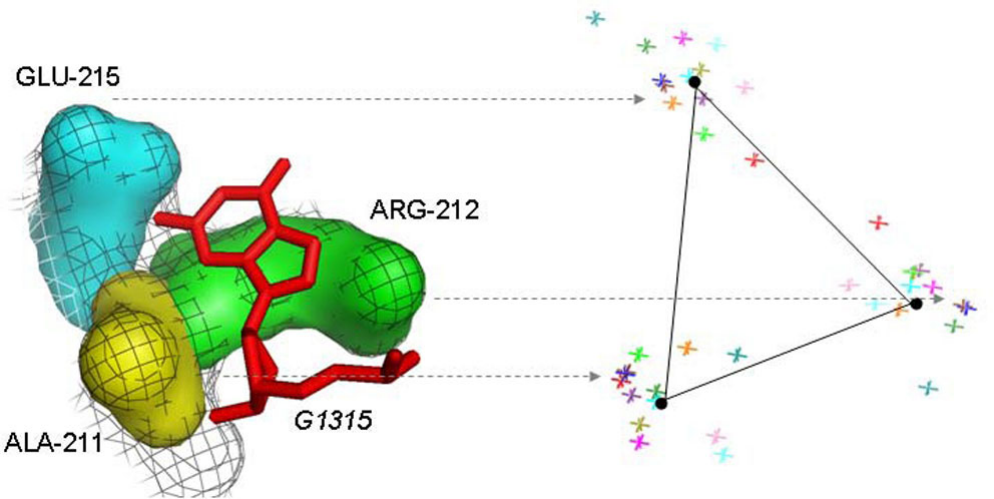
**The tripod site formed by G1315, within the kink-turn KT1311/1338, with L32e**. The three amino acids forming the tripod are colored with different colors, the extruded nucleotide in red, while the black mesh corresponds to the protein contact surface with the nucleotide. Few observed samples show that knobs are clustered around three vertices of a triangle with similar angles and distances.

Our analysis of these interactions revealed an interesting conformation common to 3 of them: the extruded nucleotide mainly interacts with three aminoacids of the protein; these aminoacids form a binding site characterized by three knobs disposed as vertices of a triangle with similar angles and sides. In the following we refer to this conformation as *tripod*. A tripod conformation is observed for the following interaction sites: G1315 (KT1311/1338) with L32e, A1150 (KT1146/1212) with L10, and A96 (KT92/77) with L29. In Figure 4 (on the left) a sample tripod is shown.

The characteristic shape of these binding sites suggested a possible recurrent conformation also on protein interfaces with single extruded nucleotides not belonging to kink-turns. Thus we searched for tripods on the entire surfaces using as query a fingerprint derived from the observed tripods in kink-turns. The search method is described in the Methods section. The results of the automatic search reveal a major presence of this site among protein interactions with single extruded nucleotides, belonging to either bulges, internal loops, junctions, or other non-helical regions.

Figure 4 (on the right) shows all tripods found in HM 50S after superimposition, where each knob is represented by a star. As can be seen, the points cluster in three groups corresponding to the vertices of an almost equilateral triangle. The knobs form a pocket that accommodate the extruded base. The search for tripods was run on the three ribosomes analyzed in section 3; Table 4 reports the whole set of instances. Not only this conformation is common to the discovered 27 instances, these tripods also make similar interactions, showing a preference towards purines (A:11 instances, G: 10, U: 4, C: 2). Although the majority of interactions are non-polar and van der Waals, most of tripods make also hydrogen bonds with the nitrogenous bases (see Table 4). Specifically non-polar and van der Waals contacts typically occur below the base plane, while H-bonds always interest the knobs of the tripod, being disposed in the base plane where the donor-acceptor geometry is optimal.

**Table 4:** Tripod instances. The tripods identified on protein interfaces within the three ribosomes are here reported.

Tripods							
	**NT No.**	**Base**	**AA_1_**	**AA_2_**	**AA_3_**	**Protein**	**P-R-B**
							
1S72	700^H^	A	Gln-113	Arg-115	Glu-71	L15	2-4-11
	2368	A	Arg-13	Asp-19	Leu-106	L18	2-2-10
	2815^H^	G	Arg-102	Lys-80	Tyr-104	L13	2-4-11
	746^H^	A	Glu-42	Asn-44	Leu-65	L18e	0-2-7
	96^H^	A	His-4	Glu-7	Gln-6	L29	0-2-7
	659^H^	A	Lys-63	Leu-65	Glu-42	L18e	3-6-12
	1315^H^	G	Arg-212	Ala-211	Glu-215	L32e	1-5-9
	1359	U	Ala-68	Gly-66	His-69 (Ser-63)	L4	1-4-8
	452^H^	G	Gln-178	Arg-182	Ala-181	L4	0-2-5
	327^H^	A	Lys-149	Gln-151	Asn-206	L4	3-6-10
	1356^H^	A	Arg-130	Arg-138	Lys-136	L32e	2-5-8
	308	U	Arg-97	Arg-52	Ser-94	L24	2-7-9
	1150	A	Thr-65	Lys-16	Arg-69	L10	0-7-7
							
2AWB	144	A	Met-1	Arg-3	Glu-4	L23	0-11-12
	2286 ^H^	G	Thr-23	Asn-25	Lys-36	L33	1-4-16
	1820	U	Arg-176	Ala-197	Met-200	L2	2-5-10
	2305 ^H^	U	Met-37	Arg-132	Arg-149	L5	0-3-11
	636 ^H^	G	Thr-74	Glu-76	Arg-126	L15	2-2-8
	2250	G	Val-80	Arg-81	Met-82	L16	0-3-12
	1156	A	Arg-47	Gln-51	Arg-54	L20	1-2-8
	1252 ^H^	G	Tyr-31	Arg-32	Gln-36	L20	1-3-10
							
1FJF	7 ^H^	G	Lys-92	Lys-121	Thr-120	S5	0-2-8
	1347 ^H^	G	Arg-10	Lys-11	Arg-107	S9	1-4-16
	691 ^H^	G	Asn-26	Gly-52	Lys-55	S11	2-3-6
	562 ^H^	C	Arg-15	Glu-16	Val-18	S12	1-10-5
	1317	C	Phe-16	Arg-19	Val-18	S14	2-4-9
	958 ^H^	A	Lys-55	Thr-77	Gly-54	S19	1-2-10
				18			

Each instance is defined by the binding nucleotide (NT), its triplet of amino acids, the protein such triplet belongs to, and P-R-B ratio. Nucleotides making H-bonds are denoted by the H apex. The three knobs interacting with U1359 include atoms from a forth residue reported in brackets.

The tripods show conserved three-dimensional structure, nucleotide preference, and similar interaction pattern; these considerations together suggest this binding site as a recurrent RNA recognition motif. This novel motif gives a good starting point to provide a better structural characterization and hopefully an effective prediction method for protein-RNA interactions.

## Conclusion

Aim of this paper was to dissect protein-RNA interaction mechanisms within the ribosome. The high conformational diversity of protein interfaces with rRNA, following the high diversity in folds of r-proteins, is confirmed by this study. However, when focusing on restricted areas some geometric and physico-chemical patterns could be detected.

Specifically, we explored the role of RNA structural motifs in protein-RNA interactions. We quantitatively showed the preference for protein interfaces to interact with structural RNA motifs by comparing the composition in terms of motifs and helical regions of RNA interfaces with that of the complete ribosomal surface.

We detected similar binding sites for extruded RNA bases leading to the definition of a novel protein structural motif, the tripod, characterized by the presence of three amino acids forming three knobs on the surface at the vertices of triangle, with approximately same sides.

Among RNA motifs tetraloops show the highest percentage of interactions with ribosomal proteins. Protein contact surfaces with tetraloops showed high conformational diversity making binding site characterization tough. By studying the localization of the contacts, preferred contact areas were detected; furthermore, a consensus interaction pattern was identified based on physico-chemical properties of the amino acids composing the interfaces.

Future work will exploit the results of this work to provide accurate computational methods to compare protein-RNA interactions in large datasets and possibly tackle the challenging problem of predicting protein-RNA interactions.

## Methods

### Interaction Maps

Interaction maps are basically a map in 2D space of the positions of the atoms of all TL_*P *_-CS after they are brought into a common reference frame. The reference frame is defined by a chosen tetraloop; all interfaces are transformed after the superposition of the tetraloops to the chosen reference. For the interaction map, we resorted to a polar representation of protein interfaces. Since the backbone of a tetraloop typically forms a curve resembling a semi circumference, the whole structure has a semi spherical shape. Thus, we projected the protein surface points on a sphere with fixed radius and associated to each point two coordinates, the zenit, *θ*, and azimut, *φ*; angles. The interaction map is a discretized representation of these points on a grid. The granularity of the cells of the grid depends on the quantization step applied to (*θ*, *φ*) values. The step has been determined as the highest value so that each atom of a single contact surface falls on a different cell. This polar coordinates based representation gives the ideal framework to design an interaction pattern easily understandable and, possibly, comparable.

### Tripods: fingerprint and search

The first instances of tripods have been derived by visual inspection of extruded nucleotides, in particular within RNA kink-turns. From this limited set of observed instances, we derived a geometric fingerprint defined by a triplet of points and their coordinates in the 3D space. The fingerprint defines a triangle with sides *l*_1, _*l*_2_, *l*_3 _∈ (7,10) Å and angles *α*_1_, *α*_2_, *α*_3 _∈ (40°, 70°). The fingerprint can thus be seen as a triplet of knobs, whose coordinates are the averages of coordinates of the knobs of the true positive tripods.

**Figure 5:**
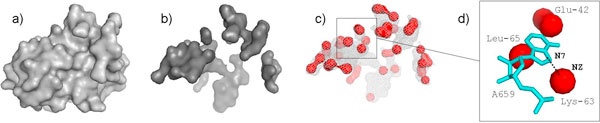
**The tripod search procedure: (a) given a protein surface, (b) the contact surface is extracted and (c) within it the knobs (highlighted in red) are isolated by detecting the convex areas**. Each triplet of disjoint knobs is then matched with the fingerprint. The extracted tripod is shown in (d).

We use this fingerprint to search for tripods on the interfaces of r-proteins. The search method takes in input a protein contact surface and extracts the atoms belonging to its convex patches using the method presented in [27]. The extracted atoms are those that form convex bulges on the protein 3D surface and thus they constitute the knobs of the contact surface. We compute a set of candidate tripods by enumerating all triplets of disjoint knobs, i.e. belonging to different amino acids, that interact with the same nucleotide. For each candidate, we determine its best matching with the fingerprint, i.e. the one-to-one correspondence between the two triplets of points that minimizes the RMSD after rigid superimposition. Only candidates with an RMSD below a given threshold are retained. Tripods are furthermore filtered using the P-R-B ratio of the nucleotide they bind. Precisely if *B *≥ *R*, *R *≥ *P*, *B *>*P *then the candidate is accepted in the final list. These relations, which are empirically derived from the observed instances proved to be a good criterion to filter out a large number of false positives, while preserving the correct instances. Figure 5 summarizes the search procedure. The final list presented in Table 4 was derived from the set produced by the search procedure aided by visual inspection.

## Competing interests

The authors declare that they have no competing interests.

## Authors' contributions

All authors contributed in the planning of the study and statistical analysis of the data. GC and Cl.G extracted the data and performed the analysis of 3D interfaces with tetraloops. GC analyzed tripod conformations and design the search procedure. Co.G coordinated the study and drafted the manuscript. GC and Co.G contributed to finalize the manuscript. All authors read and approved the final manuscript.
